# Hydrogen inhibits the proliferation and migration of gastric cancer cells by modulating lncRNA MALAT1/miR-124-3p/EZH2 axis

**DOI:** 10.1186/s12935-020-01743-5

**Published:** 2021-01-22

**Authors:** Baocheng Zhu, Hengguan Cui, Weiqiang Xu

**Affiliations:** 1 Qingpu Branch of Zhongshan Hospital Affiliated to Fudan University, Shanghai, China; 2 Qingpu Branch of Zhongshan Hospital, Fudan University, 1158 Park East Road, Qingpu District, Shanghai, 201700 China

**Keywords:** Hydrogen, Gastric cancer, LncRNA MALAT1, miR-124-3p, EZH2, Proliferation, Migration

## Abstract

**Background:**

Gastric cancer is one of the most prevalent and deadly malignancies without efficient treatment option. This study aimed to investigate the effect of hydrogen gas on the behavior of gastric cancer cells.

**Methods:**

Gastric cancer cell lines MGC-803 and BGC-823 were treated with or without H_2_ /O_2_ gas mixture (66.7%:33.3% v/v). Proliferation and migration were assessed by MTT and scratch wound healing assays respectively. The expression of lncRNA MALAT1, miR-124-3p, and EZH2 was analyzed by real-time quantitative PCR and/or western blot. Tumor growth was estimated using xenograft mouse model.

**Results:**

H_2_ gas significantly inhibited gastric tumor growth in vivo and the proliferation, migration, and lncRNA MALAT1 and EZH2 expression of gastric cancer cells while upregulated miR-124-3p expression. LncRNA MALAT1 overexpression abolished all the aforementioned effects of H_2_. LncRNA MALAT1 and miR-124-3p reciprocally inhibited the expression of each other. MiR-124-3p mimics abrogated lncRNA MALAT1 promoted EZH2 expression and gastric cancer cell proliferation and migration.

**Conclusions:**

These data demonstrated that H_2_ might be developed as a therapeutics of gastric cancer and lncRNA MALAT1/miR-124-3p/EZH2 axis could be a target for intervention.

## Background

Gastric cancer is the sixth most-common cancer with over a million new cases worldwide in 2018 and causes second most mortalities among malignancies [1]. While *Helicobacter pylori* infection is the most prominent cause of gastric cancer, many nutritional and life style factors, such as drinking, smoking, physical activity, overweight, intake of fruit and vegetable, are also significantly associated with the development of gastric cancer [2]. Genetically, the main oncogene of gastric cancer is CDH1 (E-cadherin) as many pathogenic variants are associated with familial diffuse-type gastric cancer [3, 4]. However, many other genes including *MSH2*, *PMS2*,, *BRCA1, PALB2, CTNNA1, and ATM*, have been identified to increase the risk of gastric cancer [5–12].

Long noncoding RNAs (lncRNAs) are transcripts larger than 200 base-pairs and similar to mRNA biochemically and structurally but do not code for protein [13]. A large number of lncRNA have been implicated in different aspects of cancer biology [14]. LncRNA MALAT1 (metastasis associated lung adenocarcinoma transcript 1) has been shown to promote the proliferation and migration of cancer cells, epithelial-mesenchymal transition, and metastasis of many cancer types but recently found to function as a tumor suppressor in breast and colorectal cancers [16]. LncRNA MALAT1 enhanced the stemness, proliferation, migration, invasion, and drug resistance of gastric cancer cells [17–20].

Although hydrogen gas was found effective in treating mouse squamous cell carcinoma and thought it might be used to treat other cancer [21], its clinical application was not widely explored until 2007 Ohsawa et al. demonstrated that hydrogen gas selectively eliminated the hydroxyl radical and attenuated focal ischemia and reperfusion caused oxidative stress and brain injury [22]. Increasing evidence showed the potential of hydrogen gas in preventing and relieving different cancers [23]. Drinking hydrogen-rich water for 6 weeks reduced reactive oxygen metabolites in the blood, maintained blood oxidation potential, improved the quality of life of malignant liver cancer patients after radiotherapy [24]. Daily inhalation of hydrogen for 3 month resulted in the shrinkage of metastatic gallbladder cancer and improve of quality of life [25]. This study aims to explore the effects of hydrogen gas on gastric cancer cells and the underlying molecular mechanism.

## Materials and methods

### Cell culture

Human gastric cancer cell lines MGC-803 and BGC-823 were purchased from the Type Culture Collection of the Chinese Academy of Sciences (Shanghai, China). Cells were cultured at 37 °C and 5% CO_2_ in Dulbecco’s Modified Eagle Medium (DMEM) supplemented with 10% fetal bovine serum (FBS), 100 U/ml of penicillin and 100 µg/ml of streptomycin (All from ThermoFisher, (Shanghai, China).

Hydrogen/oxygen gas mixture (66.7%:33.3% v/v) was produced with a Hydrogen/Oxygen Generator (Asclepius Meditec, Shanghai, China). Hydrogen treatment was executed in an adjustable three gas cell culture incubator (Puhe Bio, Wuxi, China). MALAT1 overexpression vector (GenScript, Nanjing, China) and miR-124-3p mimics (GeneCopoeia, Guangzhou, China) were transfected using Lipofectamine 3000 (ThermoFisher, Shanghai, China).

### MTT assay

Seeded 5000 MGC-803 or BGC-823 cells per well in 96 well plates and cultured at 37 °C overnight before treated with or without hydrogen gas for 24 h. Added 20 µl of 5 mg/ml MTT (3-(4,5-dimethylthiazol-2-yl)-2,5-diphenyltetrazolium bromide) to each well and incubated at 37 °C for 4 h. The medium was carefully removed and 100 µl of MTT solvent (4 mM HCl and 0.1% Nondet P-40 (NP40) in isopropanol) was added to every well. The plates were incubated at room temperature with orbital shaking for 15 min before read at 590 nm.

### Would healing scratch assay

After MGC-803 and BGC-823 cells in 24 well plates reached confluency, the cells were treated with 0.2 mg/ml mitomycin C for 3 min. The center of the wells were scratched with a blue tip and photographed. The plates then cultured at 37 °C for 12 h and photographed. The gaps were measured with ImageJ (NIH, Bethesda, MD). The migration rate was calculated as (gap distance at 0 h − gap distance at 24 h)/gap distance at 0 h *100.

### Quantitative real‐time polymerase chain reaction (RT-qPCR)

Total RNA was extracted from using MiniBEST Universal RNA Extraction Kit (TaKaRa, Beijing, China) following manufacturer’s manual. First strand cDNA was synthesized from 0.5 µg total RNA using Invitrogen SuperScript III Reverse Transcriptase kit (ThermoFisher, Shanghai, China). Noncoding small RNAs were first poly (A) tailed with *E. coli* Poly(A) Polymerase (M0276, NEB, Ipswich, MA) and then reverse transcribed using Moloney Murine Leukemia Virus (M-MuLV, MMLV) Reverse Transcriptase (M0253, NEB) with GTCGCAGTGCAGGGTCCGAGGTGGCGATTTTTTTTTTTTTTTTTTTTTTT(A/G/C)(A/G/T/C) as primer. Real-time RT-PCR amplication was carried on an ABI StepOne Plus (Applied Biosystems, Foster City, CA) using SYBR Premix Ex TaqTM kit (Takara, Beijing, China). The PCR primers were TGCTGTGTGCCAATGTTTCG and CAGCTGCCTGCTGTTTTCTG for MALAT1, CTGCTTCCTACATCGTAAGTGCAA and TTGCTCCCTCCAAATGCTGGT for enhancer of zeste 2 polycomb repressive complex 2 subunit (EZH2), CTGAGGAGCAGCTTCAGTCC and GAGTAGCCATTGTCCACGCT for β-catenin (CTNNB1), and CCGAGAATGGGAAGCTTGTC and AAGCACCAACGAGAGGAGAA for glyceraldehyde 3-phosphate dehydrogenase (GAPDH). TAAGGCACGCGGTGAATGC for miR-124-3p and CGCAAGGATGACACGCAAATTC for U6 with universal reverse primer GTGCAGGGTCCGAGGT. The relative gene expression was calculated using 2^− ΔΔCt^ method with GAPDH as internal control for lncRNA MALAT1 and EZH2 while U6 for miR-124-3p.

### Western blot

MGC-803 and BGC-823 cells were lyzed with RIPA lysis buffer (1% v/v NP-40, 20 mM Tris-HCL pH 7.4, 5 mM sodium pyrophosphate, 5 mM EDTA) supplemented with protease and phosphatase inhibitor cocktail (Millipore Sigma, Burlington, MA). Total protein samples (40 µg) were resolved on 8% SDS-PAGE gels and transferred onto PVDF membranes. The membranes were blocked in 5% nonfat milk in TBST (50 mM Tris, pH 7.5; 150 mM NaCl; 0.1% Tween 20) for 30 min, incubated with primary antibodies at 4 °C overnight, washed and incubated with proper horseradish peroxidase conjugated goat against mouse or rabbit IgG antibodies (Jackson ImmunoResearch, West Grove, PA) at room temperature for 60 min before visualized with enhanced chemiluminescence (ECL) reagents (Pierce, Rockford, IL). The primary antibodies used were anti-EZH2 (05-1319, Millipore Sigma) and anti-β-actin (A5441, Millipore Sigma).

### Mouse xenograft gastric cancer models

Animal protocol was approved by Institutional Animal Care and Usage Committee of Zhongshan Hospital Affiliated to Fudan University (ZHFU20200374). Female BALB/c nude mice (5 weeks old) were purchased from Cavens Laboratory Animal Inc (Changzhou, China) and acclimatized for a week. BGC-823 cells (1 × 10^6^) stably transfected with lncRNA MALAT1 or miR-124-3p were subcutaneously injected into the flank of a nude mouse (n = 7). Three days later, mice were exposed to hydrogen/oxygen mixture (2:1 vol:vol) for 2 h daily. All mice were sacrificed 5 weeks after inoculation.

### Statistical analyses

All experiments were performed 3 times with triplicates. Statistical analyses were performed using GraphPad Prism 6 (San Diego, CA). The differences among treatments were assessed with analysis of variance followed by Bonferroni tests. A p value less than 0.05 was considered statistical significant.

## Results

### Hydrogen inhibited the proliferation and migration of gastric cancer cells

**Fig. 1:**
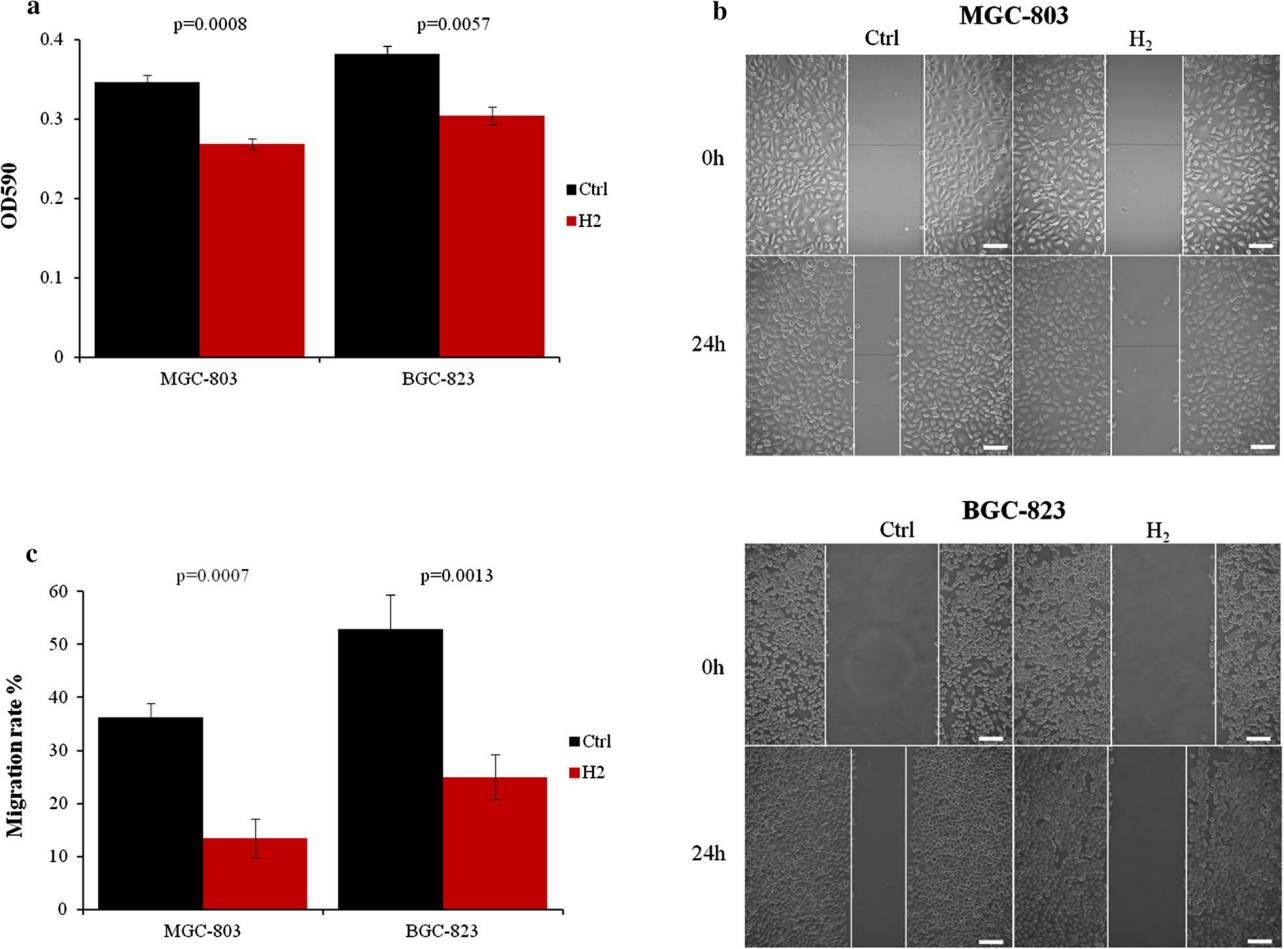
Gastric cancer cell proliferation and migration were inhibited by H_2_ gas. **a** MGC-803 and BGC-823 cells were cultured with or without H_2_ for 24 h and assessed by MTT assay. **b** MGC-803 and BGC-823 cell migration was assayed by scratch wound healing assay. **c** Quantitative analysis of migration data. Scale bar: 25 µm. Ctrl, Normal culture conditions. *p < 0.01 compared to Ctrl

As hydrogen gas has been shown to inhibit cancer progression [25] and lung cancer cell proliferation [26], we first examined whether it could impact the proliferation of gastric cancer cells. After 24 h exposure to hydrogen gas, the viable MGC-803 and BGC-823 cells were reduced more than 20% compared to normally cultured ones (Fig. 1a). Moreover, H2 gas drastically reduced the motility of MGC-803 cells by about 60% and BGC-823 cells by more than 50% (Fig. 1b, c).

### Hydrogen gas inhibited the expression of MALAT1 and EZH2

**Fig. 2:**
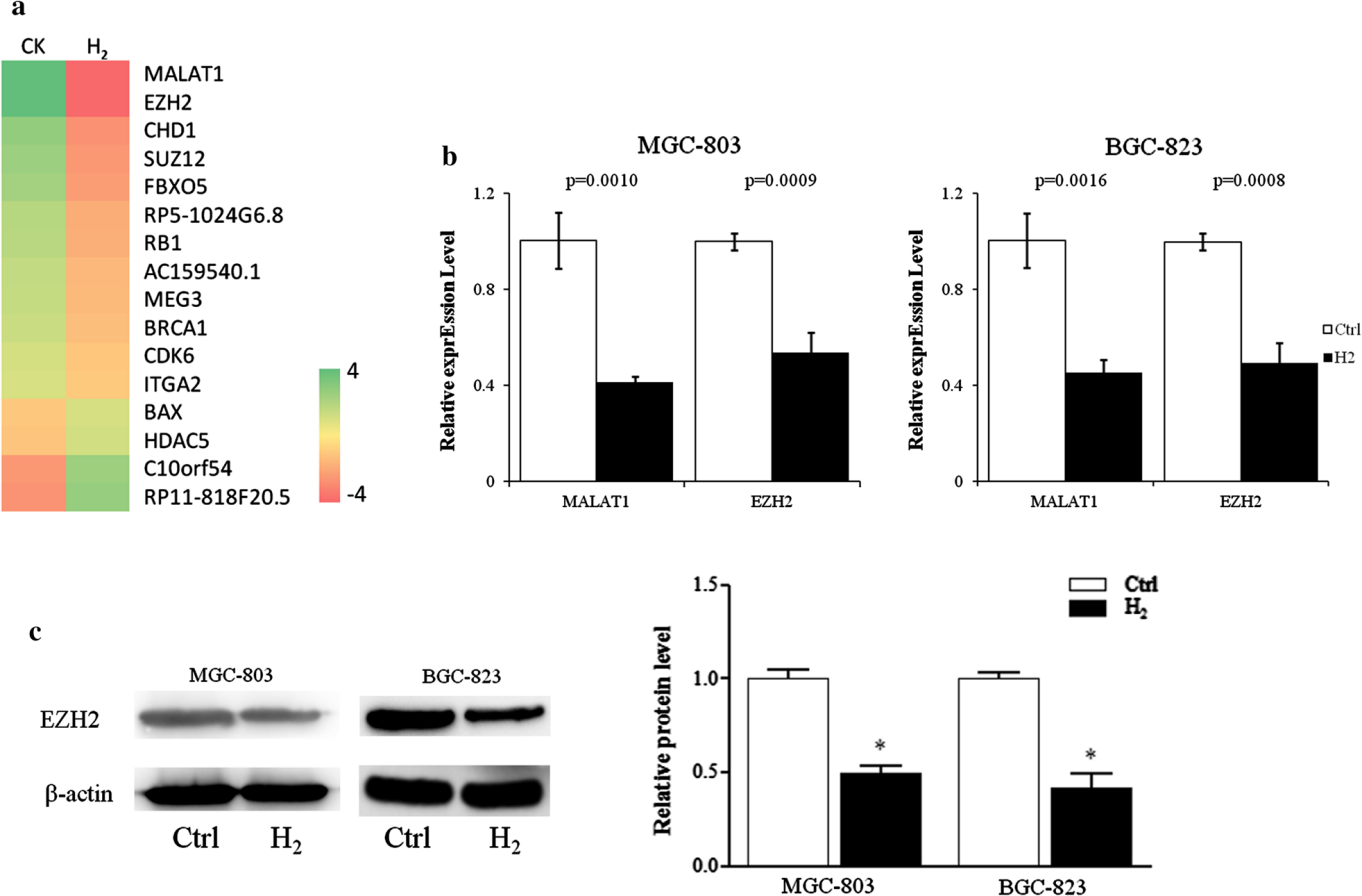
The expression of lncRNA MALAT1 and its target genes in gastric cancer cells was inhibited by H_2_. **a** Heatmap of genes differently expressed in MGC-803 cells cultured with or without H_2_. **b** Transcript levels of lncRNA MALAT1, EZH2, were analyzed by quantitative real-time PCR. **c** Protein levels of EZH2 were assessed by immunoblot. CK & Ctrl, Normal culture conditions. *p < 0.01 compared to Ctrl

Microarray analysis identified that H_2_ gas significantly inhibited the expression of lncRNA MALAT1 and transcription factor EZH2 of MGC-823 cells (Fig. 2a). Real-time quantitative PCR confirmed that 24 h H_2_ exposure reduced the RNA transcript levels of MALAT1 and EZH2 about 50% in both MGC-803 and BGC-823 cells (Fig. 2b). The protein levels of EZH2 of MGC-803 and BGC-823 cells treated with H_2_ were reduced more than 50% compared to control cells (Fig. 2c).

### H_2_ inhibited EZH2 expression through downregulating LncRNA MALAT1

**Fig. 3:**
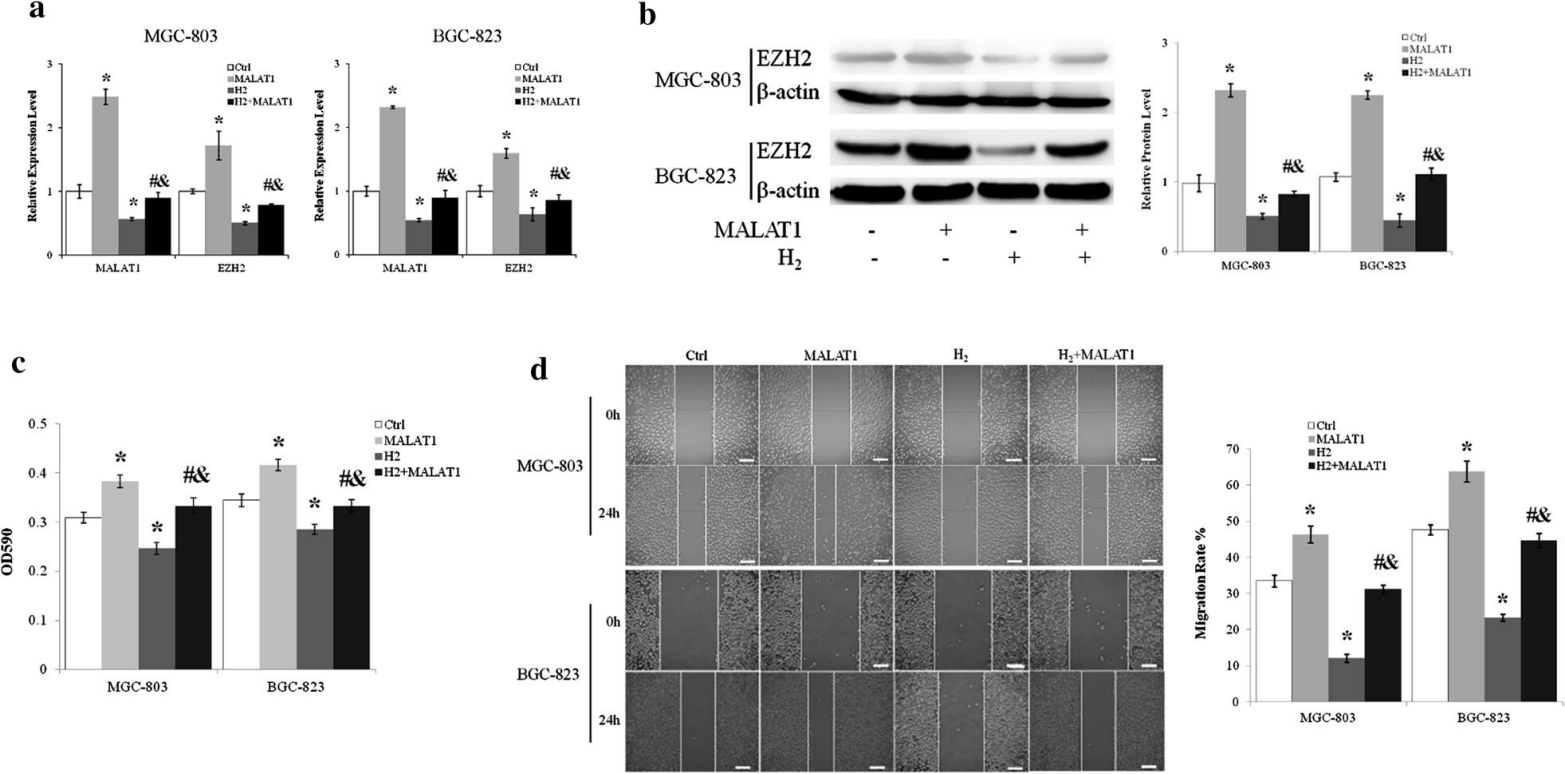
H_2_ inhibited gastric cancer cell proliferation and migration through regulating lncRNA MALAT1. **a** Transcript levels of lncMALAT1 and EZH2 of MGC-803 cells were measured by quantitative real-time PCR. **b** Protein level of EZH2 was assessed by immunoblot. **c** MGC-803 and BGC-823 cells transfected with or without lncRNA MALAT1 expression vector were cultured with or without H_2_ for 24 h and assessed by MTT assay. **d** Scratch wound healing assay was used to examine the effects of H_2_ and lncRNA MALAT1 on MGC-803 and BGC-823 cell migration. Scale bar: 25 µm. Ctrl, Normal culture conditions. *p < 0.01 compared to Ctrl; ^#^p < 0.01 compared to MALAT1; ^&^p < 0.01 compared to H_2_

Overexpressing MALAT1 upregulated the mRNA (Fig. 3a) and protein (Fig. 3b) levels of EZH2 in MGC-803 and BGC-823 cells. Furthermore, LncRNA MALAT1 abrogated the inhibition of EZH2 expression by H_2_ (Fig. 3a, b).

### LncRNA MALAT1 relieved the inhibitory effect of H_2_ on gastric cancer cell proliferation and migration

As previously showed that H_2_ gas inhibited the proliferation and migration of MGC-803 and BGC-823 cells, we next examined the roles of LncRNA MALAT1 on the effects of H_2_. LncRNA MALAT1 overexpression resulted in about 25% increase of MGC-803 and BGC-823 cell proliferation (p < 0.01 MALAT1 vs. Ctrl) and abolished the inhibition of proliferation imposed by H_2_ (p < 0.01 for MALAT1 + H_2_ vs. MALAT1 and MALAT1 + H_2_ vs. H_2_) (Fig. 3c). Overexpression of LncRNA MALAT1 increased MGC-803 and BGC-823 cell migration by about 40% (p < 0.01 MALAT1 vs. Ctrl) (Fig. 3D). Moreover, MALAT1 abrogated the inhibitory effect of H_2_ gas on the migration of MGC-803 gastric cancer cells (p < 0.01 for MALAT1 + H_2_ vs. MALAT1 and MALAT1 + H_2_ vs. H_2_) (Fig. 3d).

### miR-124-3p expression in gastric cancer cells was regulated by H_2_ and lncRNA MALAT1

**Fig. 4:**
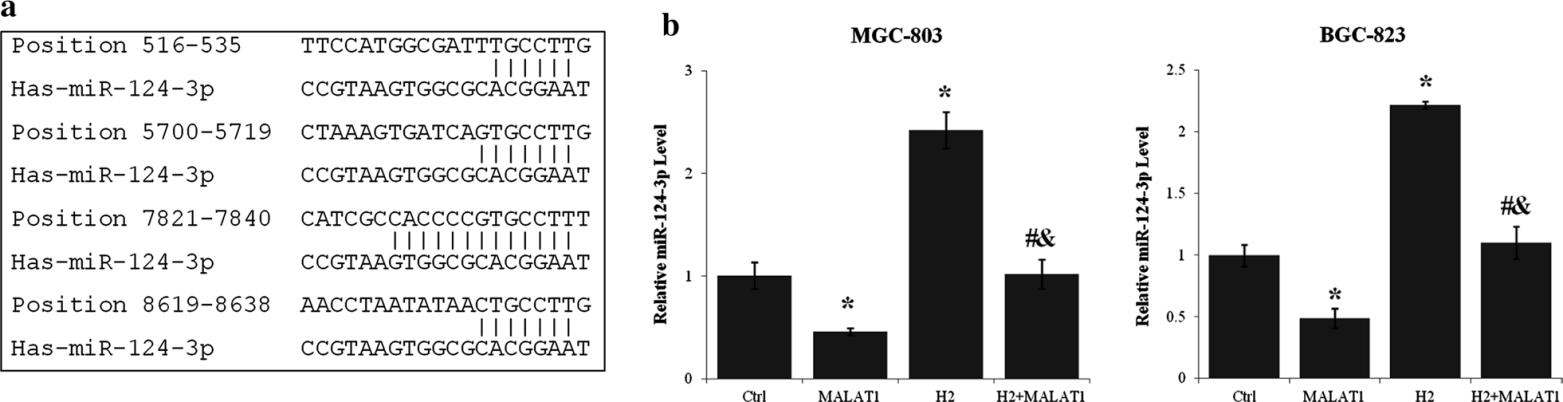
LncRNA MALAT1 mediated the effects of H_2_ on miR-124-3p expression in gastric cancer cells. **a** The binding sites of miR-124-3p within lncRNA MALAT1 transcript sequence predicted using LncBase Predicted v.2 of Diana Tools. **b** The expression level of miR-124-3p in MGC-803 and BGC-823 cells transfected with or without lncRNA MALAT1 expression vector and cultured with or without H_2_ for 24 h was assayed by quantitative real-time PCR. *p < 0.01 compared to control; ^#^p < 0.01 compared to MALAT1; ^&^p < 0.01 compared to H_2_

Using LncBase Predicted v.2 (http://carolina.imis.athena-innovation.gr/diana_tools), has-miR-124-3p was identified as a regulator and target of lncRNA MALAT1. Four strong miR-124-3p binding sites (Fig. 4a) and many other imperfect binding sites of miR-124-3p were identified in MALAT1. The miR-124-3p level of MGC-803 and BGC-823 cells was increased more than 100% by H_2_ treatment (p < 0.01, H_2_ vs. Ctrl) (Fig. 4b). On the other hand, lncRNA MALAT1 overexpression reduced miR-124-3p level more than 40% (p < 0.01 MALAT1 vs. Ctrl). Moreover, lncRNA MALAT1 and H_2_ gas antagonized the effect of each other on the expression of miR-124-3p (p < 0.01 for MALAT1 + H_2_ vs. MALAT1 and MALAT1 + H_2_ vs. H_2_) (Fig. 4b).

### LncRNA MALAT1 upregulated EZH2 expression through inhibiting miR-124-3p

**Fig. 5:**
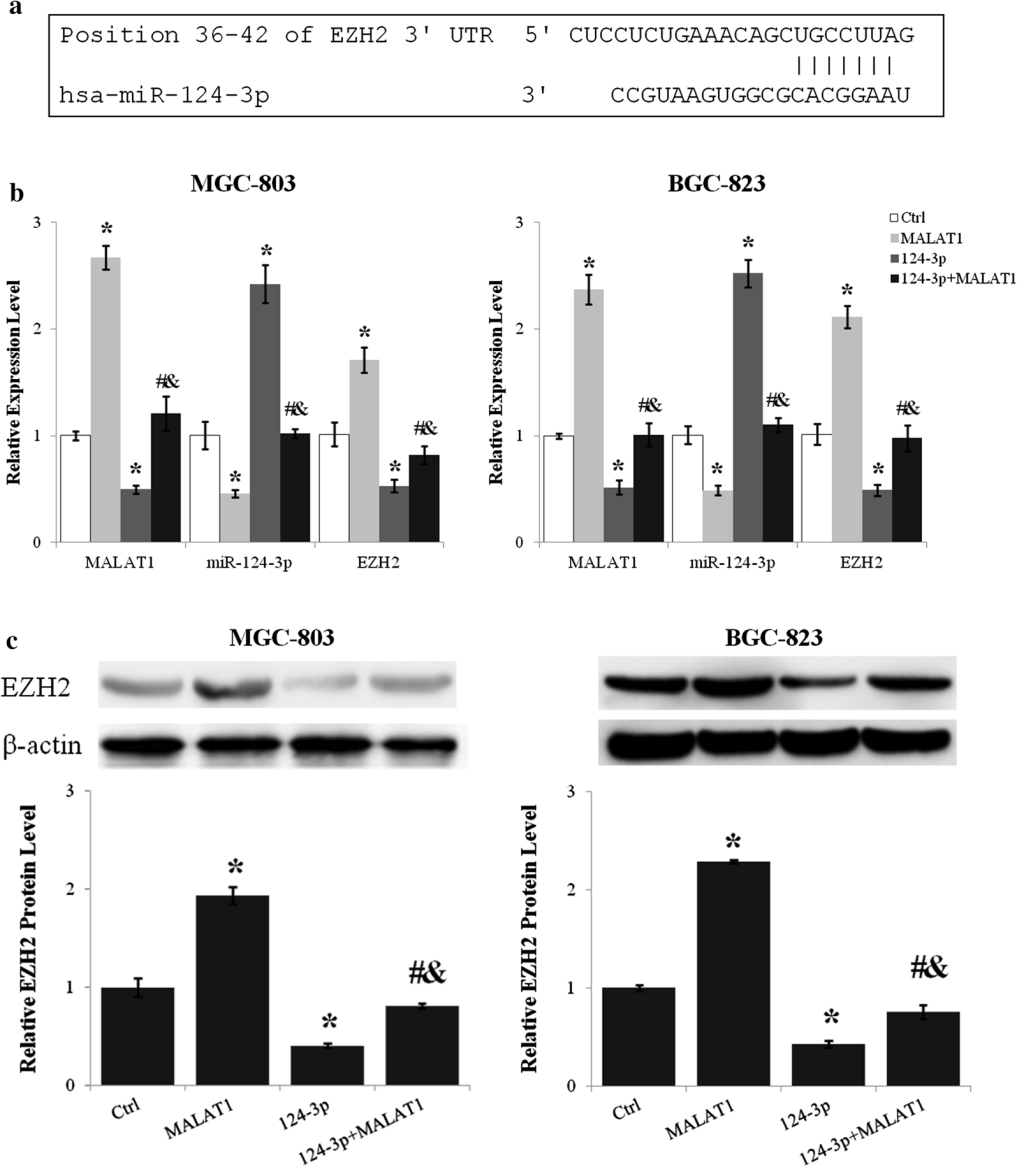
The upregulation of EZH2 by lncRNA MALAT1 was antagonized by miR-124-3p. **a** The miR-124-3p binding site in EZH2 3’UTR predicted using TargetScan. **b** The expression levels of lncRNA MALAT1, miR-124-3p, and EZH2 after lncRNA MALAT1 and/or miR-124-3p overexpression were analyzed by quantitative real-time PCR. **c** The effects of lncRNA MALAT1 and miR-124-3p on EZH2 protein level were examined by western blot. Ctrl, Normal culture conditions. *p < 0.01 compared to control; ^#^p < 0.01 compared to MALAT1; ^&^p < 0.01 compared to 124-3p

As miR-124-3p was regulated by lncRNA MALAT1 and EZH2 was identified as a direct target of miR-124-3p (Fig. 5a), the effect of miR-124-3p on the regulation of EZH2 expression by lncRNA MALAT1 was assessed. Transfection of miR-124-3p mimics reduced EZH2 mRNA level about 50% (p < 0.01, miR-124-3p vs. Ctrl) and suppressed the upregulation of EZH2 by lncRNA MALAT1 (p < 0.01 for miR-124-3p + MALAT1 vs. MALAT1 and miR-124-3p + MALAT1 vs. miR-124-3p) (Fig. 5b). Moreover, lncRNA MALAT1 and miR-124-3p reciprocally inhibited the expression of one another (Fig. 5b). The protein level of EZH2 was reduced about 60% by miR-124-3p (p < 0.01, miR-124-3p vs. Ctrl) and miR-124-3p abrogated the lncRNA MALAT1 induced EZH2 upregulation (p < 0.01 for miR-124-3p + MALAT1 vs. MALAT1 and miR-124-3p + MALAT1 vs. miR-124-3p) (Fig. 5c).

### miR-124-3p abolished lncRNA MALAT1 induced proliferation and migration of gastric cancer cells

**Fig. 6:**
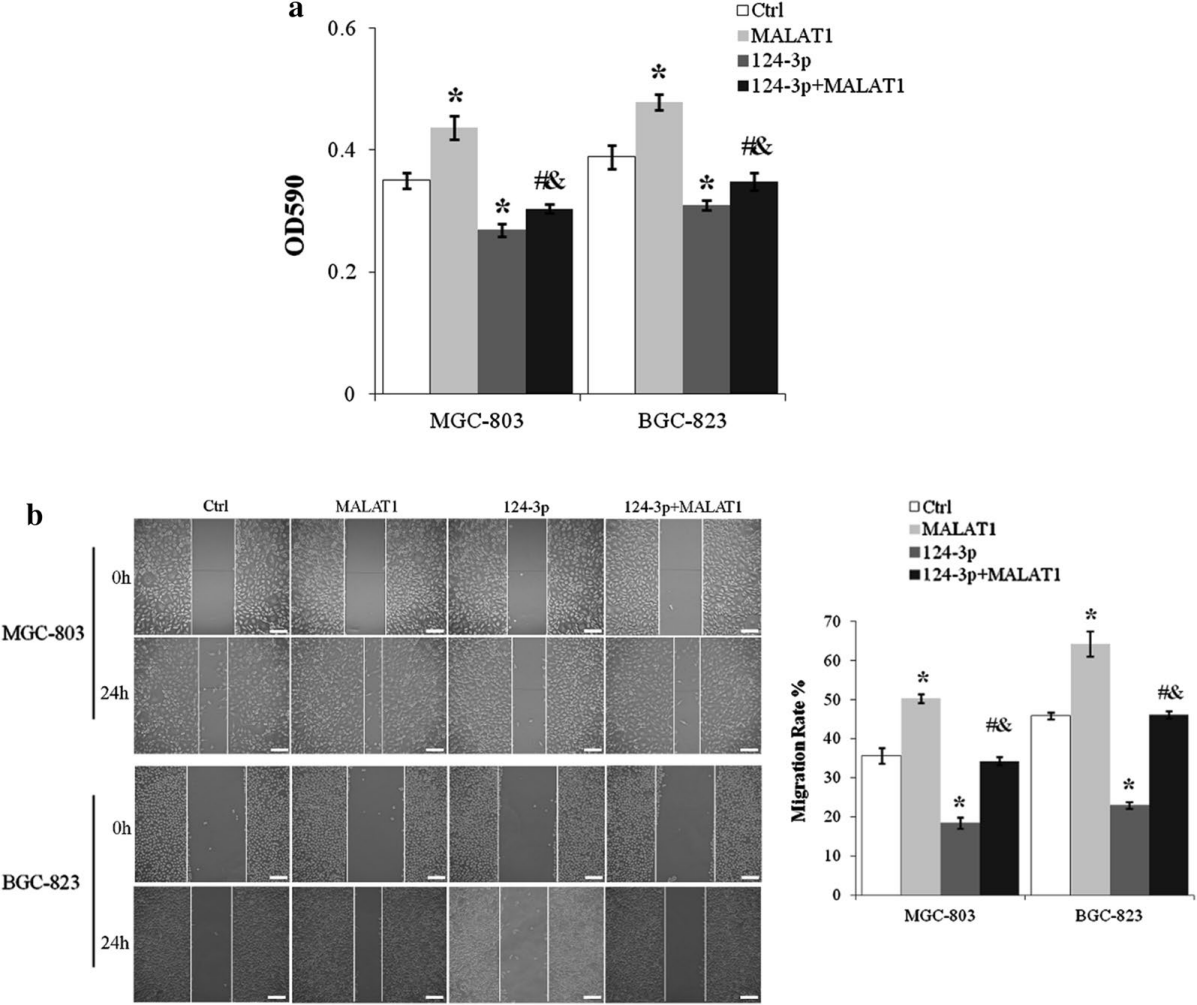
miR-124-3p blocked lncRNA MALAT1 induced proliferation and migration of gastric cancer cells. **a** MGC-803 cells transfected with lncRNA MALAT1 and/or miR-124-3p were cultured for 24 h and assessed by MTT assay. **b** The migration of MGC-803 and BGC-823 cells with lncRNA MALAT1 and/or miR-124-3p overexpression was assayed by scratch wound healing assay. Scale bar: 25 µm. Ctrl, Normal culture conditions. *p < 0.01 compared to control; ^#^p < 0.01 compared to MALAT1; ^&^p < 0.01 compared to 124-3p

Viable MGC-803 and BGC-823 cells were reduced more than 20% by miR-124-3p mimics (p < 0.01, miR-124-3p vs. Ctrl) and the promotion of MGC-803 and BGC-823 cell proliferation by lncRNA MALAT1 was inhibited by miR-124-3p (p < 0.01 for miR-124-3p + MALAT1 vs. MALAT1, and miR-124-3p + MALAT1 vs. miR-124-3p) (Fig. 6a). miR-124-3p also inhibited the migration rate of MGC-803 and BGC-823 cells by about 50% (p < 0.01, miR-124-3p vs. Ctrl) and abolished lncRNA MALAT1 induced increase of cell motility (p < 0.01 for miR-124-3p + MALAT1 vs. MALAT1 and miR-124-3p + MALAT1 vs. miR-124-3p) (Fig. 6b).

### LncRNA MALAT1 and miR-124-3p mediated the inhibition of tumor growth by H_2_

**Fig. 7:**
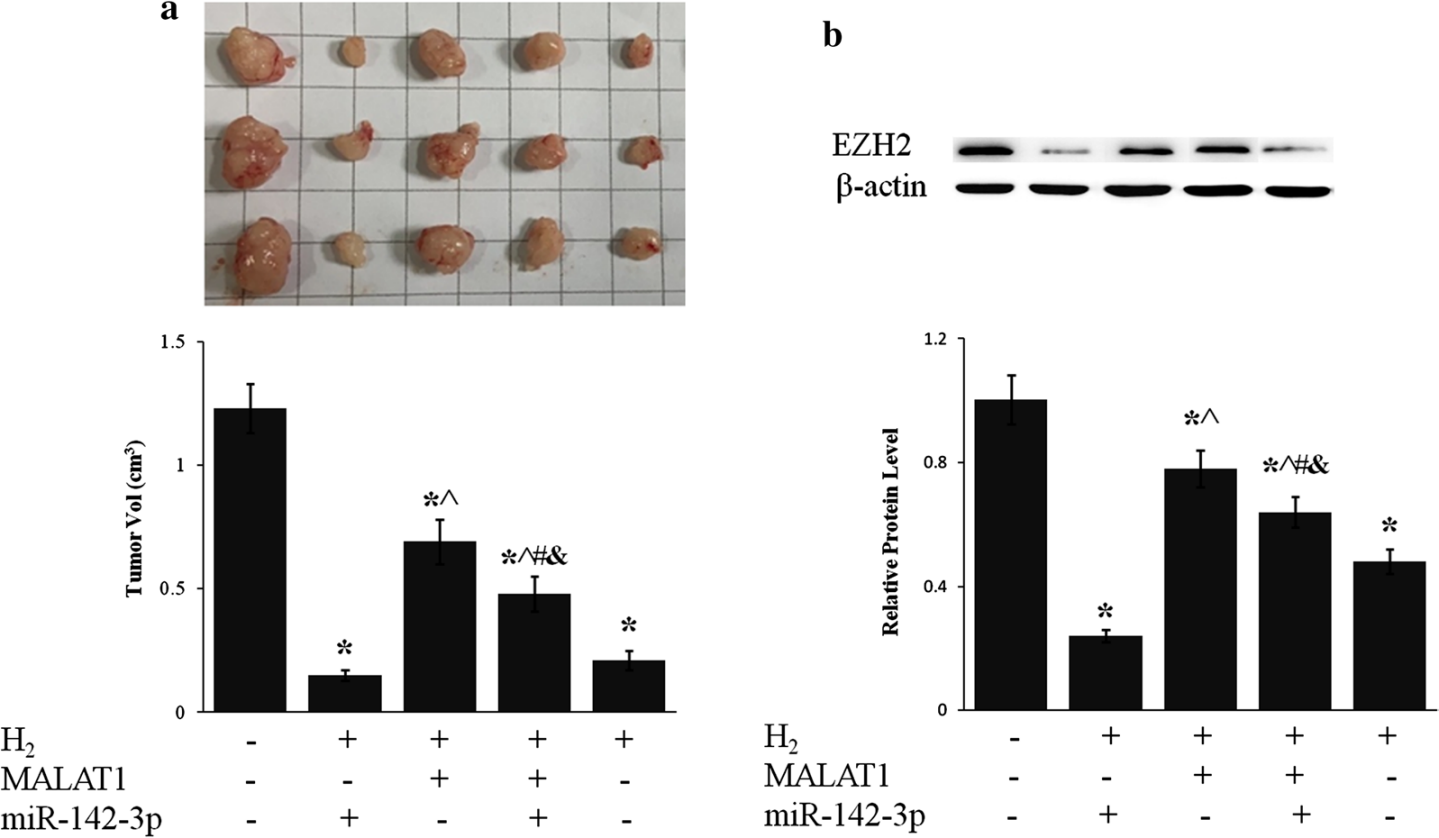
H2 inhibited gastric tumor growth via lncRNA MALAT1/miR-124-3p axis. BGC-823 cells stably expressing lncRNA MALAT1 and/or miR-124-3p were grafted into nude mice subcutaneously. Mice were then exposed to H2 for 2 h daily for 5 weeks. **a** Picture of representative tumors (upper panel) was shown and tumor size (lower panel) was measured. **b** EZH2 protein level of tumor tissue was evaluated by western blot. *p < 0.01 compared to Ctrl; ^^^p < 0.01 compared to H_2_ + 124-3p; ^#^p < 0.01 compared to H_2_ + MALAT1; ^&^p < 0.01 compared to H_2_

Inhalation of H_2_ drastically inhibited tumor growth in mouse xenografted with BGC-823 cells (p < 0.01, H_2_ vs. Ctrl) (Fig. 7), which was consistent with clinical observation of different cancers [23, 25]. Overexpression of lncRNA MALAT1 partially suppressed H_2_ caused inhibition of tumor growth whereas miR-124-3p counteracted the effect of lncR MALAT1 (Fig. 7a). EZH2 protein level of gastric tumor tissue was reduced more than 50% by H_2_ exposure, which was enhanced by miR-124-3p and suppressed by lncRNA MALAT1 (Fig. 7b).

## Discussion

H_2_ gas inhibited the proliferation and migration of MGC-803 and BGC-823 gastric cancer cells and downregulated the expression of lncRNA MALAT1and EZH2. The inhibition of EZH2 by H_2_ was relieved by lncRNA MALAT1 overexpression. LncRNA MALAT1 also alleviated H_2_ caused inhibition of the proliferation and migration of MGC-803 and BGC-823 gastric cancer cells. MicroRNA hsa-miR-124-3p was identified as a target and regulator of lncRNA MALAT1 and suppressed the expression of EZH2. H_2_ upregulated miR-124-3p expression, which was abrogated by lncRNA MALAT1 overexpression. LncRNA MALAT1 promoted proliferation and migration of MGC-803 and BGC-823 cells were abolished by miR-124-3p mimics. H_2_ exposure drastically reduced gastric cancer growth in xenograft mouse model and inhibited EZH2 expression in cancer tissue, which was enhanced by miR-124-3p and suppressed by lncRNA MALAT1.

H_2_ gas has been shown to improve many conditions by clearing detrimental ROS [27]. Inhalation of high concentration of hydrogen gas protected rat retinal ganglion cells from retinal ischemia/reperfusion (I/R) injury caused oxidative stress, inflammation, and apoptosis [28]. H_2_ inhalation ameliorated middle cerebral artery occlusion induced cerebral I/R injury evidenced by reduced 3-nitrotyrosine and 8-hydroxy-2-deoxyguanosine production, tampered inflammation, and reduced neural apoptosis [29]. PI3K/AKT1 signaling was shown to mediate the protection of H_2_ on I/R induced cardiac injuries [30]. Malignant liver cancer patients drinking hydrogen-rich water during radiotherapy significantly reduced blood hydroperoxide level and increased biological antioxidant activities, leading to improved quality of life [24]. Daily H_2_ inhalation stopped the growth of primary and metastatic tumors in a metastatic gallbladder cancer patient with the levels of tumor biomarkers returning to normal [25]. H_2_ treatment inhibited CD47 expression and induced apoptosis of A549 lung cancer cells [31]. Another study showed that SMC3 mediated the inhibition of lung cancer progression by H_2_ in in vitro study and xenograft mouse model [26]. The current study showed that H_2_ could inhibit the progression and migration of gastric cancer cells. Further studies about the effects of H_2_ on gastric cancer progression in animal models are warranted.

Long noncoding RNA MALAT1 was identified as a factor associated with metastasis of non-small cell lung cancer [32] and regulated gene expression through different mechanism [16]. LncRNA MALAT1 was overexpressed in gastric cancers compared to adjacent normal tissue and promoted gastric cancer cells proliferation, migration, and resistance to cisplatin through activating PI3K/AKT pathway [19, 33]. LncRNA MALAT1 regulated miR-30e/ATG5 expression to modulate autophagy and apoptosis of SGC7901 gastric cancer cells and conferred resistance to cisplatin [34]. MALAT1 was shown to directly bind to and stabilize Sox2 mRNA, which led to the increase of stemness and chemo-and radioresistance of gastric cancer cells [20]. The current study demonstrated that lncRNA MALAT1 promoted gastric cancer proliferation and migration through modulating miR-124-3p/EZH2 axis.

**Fig. 8:**
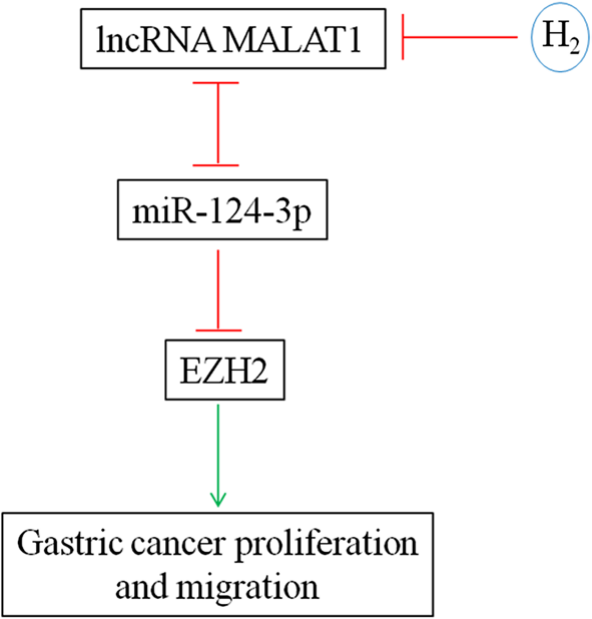
A proposed model for how H_2_ inhibits gastric cancer growth and invasion. Please refer to the text for details.  activation;  inhibition

MicroRNA miR-124-3p was significantly downregulated in gastric cancer tissues and its level was inversely associated with histological grade, TNM stage and lymph node metastasis. Moreover, low miR-124-3p expression was correlated with lower overall and disease-free survival rates [35]. ITGB3 [36] and ZEB1 [37] were found to be targeted by miR-124-3p in promoting gastric cancer proliferation and invasion. Moreover, Circular RNA circ-PVT1 upregulated ZEB1 expression by sponging miR-124-3p and enhanced paclitaxel resistance of gastric tumor and gastric cancer cells [37]. LncRNA XIST and miR-124-3p competitively regulated EZH2 expression and modulated the proliferation, migration, and invasion of laryngeal squamous cancerous cells [38]. miR-124-3p was antagonized by lncRNA MALAT1 in inhibiting the expression of Slug and metastasis of hepatocellular carcinoma [39]. This study demonstrated that H_2_ gas curbed gastric cancer growth in vivo and gastric cancer cell proliferation and migration in vitro by reducing lncRNA MALAT1 level, which in turn upregulated miR-124-3p and downregulated EZH2 expression (Fig. 8).

## Conclusions

This study demonstrated the efficacy of molecular hydrogen in inhibiting the growth of gastric cancer by reducing the proliferation and migration of gastric cancer cells, which was through downregulating lncRNA MALAT1 and polycomb-group family member EZH2 and upregulating miR-124-3p. Moreover, miR-124-3p inhibited EZH2 expression and it reciprocally repressed the expression of each other with lncRNA MALAT1. These data indicated that H_2_ should be further studied for treating gastric cancers and lncRNA MALAT1/miR-124-3p/EZH2 axis would be a novel intervention target.

## Data Availability

All data generated or analyzed during this study are included in the manuscript.

## Abbreviations

MTT: 3-(4,5-Dimethylthiazol-2-yl)-2,5-diphenyltetrazolium bromide
lncRNA: Long noncoding RNA
miRNA: MicroRNA
MALAT1: Metastasis associated lung adenocarcinoma transcript 1
EZH2: Enhancer of zeste 2 polycomb repressive complex 2 subunit
GAPDH: Glyceraldehyde 3-phosphate dehydrogenase
